# ﻿The phylogeny and taxonomy of *Upretia* (Caloplacoideae, Teloschistaceae), reveal three new species from Southwestern China

**DOI:** 10.3897/mycokeys.100.111446

**Published:** 2023-12-07

**Authors:** Lijuan Li, Yanyun Zhang, Christian Printzen, Lisong Wang, Xinyu Wang

**Affiliations:** 1 Key Laboratory for Plant Diversity and Biogeography of East Asia, Kunming Institute of Botany, Chinese Academy of Sciences, Kunming 650201, China; 2 Senckenberg Research Institute and Natural History Museum, 60325, Frankfurt am Main, Germany; 3 College of Life Science, Anhui Normal University, Wuhu 241000, China; 4 Yunnan Key Laboratory for Fungal Diversity and Green Development, Kunming Institute of Botany, Chinese Academy of Sciences, Kunming 650201, China

**Keywords:** Chemistry, Hengduan Mountains, lichen, phylogenetic analyses, zeorine type apothecia

## Abstract

Several specimens of *Upretia* from Southwest China are morphologically and phylogenetically distinct from currently recognized species in the genus. These specimens are here accommodated within a new species, *Upretiazeorina* Li J. Li & Printzen. It is characterized by an areolate to squamulose thallus with brown to blackish brown upper surface, pruinose, zeorine type apothecia, black discs, narrowly bacilliform conidia, and the production of gyrophoric acid. Two other specimens of *Upretia* from China are distinct from currently accepted species and tentatively referred to as *Upretia* sp. 1 and *Upretia* sp. 2. A key to all known species of *Upretia* is also provided.

## ﻿Introduction

The family Teloschistaceae is one of the largest families of lichenized fungi, including more than 1000 known species, and divided into four subfamilies, Brownlielloideae, Caloplacoideae, Teloschistoideae and Xanthorioideae (Gaya et al. 2012; Arup et al. 2013; Kondratyuk et al. 2015). The originally monotypic genus *Upretia* S. Y. Kondr., A. Thell & J. S. Hur was described in the subfamily Caloplacoideae. Its type species, i.e., *U.amarkantakana* (Y. Joshi & Upreti) S.Y. Kondr. & A. Thell from India is characterized by a partly pruinose, lobate to subsquamulose, olivaceous grey to brown thallus, lecanorine apothecia, small ascospores and narrowly bacilliform conidia (Kondratyuk et al. 2018). Subsequently, two more species were reported, *U.hueana* (B. de Lesd.) S. Y. Kondr. et Upreti from India and *U.squamulosa* Y. Y. Zhang & Li S. Wang from China (Zhang et al. 2019; Mishra et al. 2020).

Our field work along the collection routes of Handel-Mazzetti (1914–1915) (Zahlbruckner 1930) in Hengduan Mountains, Southwest China, yielded specimens of the family Teloschistaceae, which had polarilocular ascospores and that were phylogenetically close to the genus *Upretia*. Analyses of morphological traits and phylogenetic evidence suggest that these specimens are distinct from currently recognized species. Our Chinese specimens represented three morphospecies, of which only one was shared by multiple accessions. The latter are accommodated within a new species, i.e., *Upretiazeorina* Li J. Li & Printzen, whereas the other two specimens are tentatively referred to *Upretia* sp. 1 and *Upretia* sp. 2. A key to all the known species of *Upretia* is also provided.

## ﻿Materials and methods

### ﻿Phenotypic studies

The specimens examined are deposited in Lichen Herbarium, Kunming Institute of Botany, Chinese Academy of Sciences (**KUN**), and the Herbarium Senckenbergianum Frankfurt/M. (**FR**).

External morphological characters were studied on air-dried material under a stereomicroscope (Zeiss Stemi SV11). Anatomical features were studied using a light microscope (Zeiss Axioskop 2 plus) on transverse sections of apothecia and thalli, cut with a freezing microtome (Zeiss HYRAX KS 34) to 16–20 µm thickness and mounted in water, Lugol’s iodine solution (I) and lactophenol cotton blue (LCB).

Spot tests were conducted using the following reagents: a 10% aqueous solution of potassium hydroxide (KOH) (K), saturated aqueous solution of sodium hypochlorite NaClO (C). High performance thin layer chromatography (HPTLC) was performed in solvents A, B’ and C to detect lichen secondary metabolites (Culberson and Kristinsson 1970; Arup et al. 1993). Substances were identified according to Orange et al. (2001).

### ﻿DNA extraction, PCR and sequencing

Total DNA was extracted from specimens using the GeneOn Plant DNA Extraction Kit (GeneOn BioTech, Changchun, China) by the magnetic bead method or the DNA secure Plant Kit (Tiangen Biotech, Beijing, China). The fungal internal transcribed spacer (ITS) region of the rDNA repeat was amplified via polymerase chain reaction (PCR) using the primers ITS1F (Gardes and Bruns 1993) and ITS4A (Larena et al. 1999). PCRs were performed in 25 μL volumes using Illustra PuReTaq Ready-To-Go PCR Beads (GE Healthcare Life Sciences, Little Chalfont, Buckinghamshire, UK) containing 5 μL of DNA extract and 1 μL (10mM) of each primer; or in 25 μL reactions containing 12.5 μL 2× Taq PCR Mix (Tiangen Biotech, Beijing, China), 0.5 μL of each primer, 10.5 μL ddH_2_O and 1 μL of DNA. Cycling conditions included initial denaturation at 94 °C for 5 min, followed by 4 cycles at 94 °C for 30 s, 54 °C for 45 s, and 72 °C for 60 s, 30 cycles at 94 °C for 30 s, 48 °C for 30 s, and 72 °C for 60 s, and a final extension at 72 °C for 10 min. The PCR products were sequenced by Macrogen Europe (Amsterdam, The Netherlands) or TsingKe Biological Technology (Kunming, China).

### ﻿Phylogenetic analyses

We used nrITS sequences to construct a phylogenetic tree with more species of the subfamily Caloplacoideae (Table 1). Sequences were assembled and edited in Geneious Prime 2021.0.3 (https://www.geneious.com/). Dataset was aligned using the MAFFT v.7 online service (https://mafft.cbrc.jp/alignment/server/index.html, Katoh and Standley (2013). The final alignment included 25 taxa with 562 bp.

**Table 1.** Specimens used for the phylogenetic analyses including collection information and GenBank accession numbers for nrITS sequences. Newly obtained sequences in this study are in bold.

*Species name*	Voucher details	Country	GenBank No.
*Caloplacacerina*	Elvebakk 03-084 (TROM)	Norway	KC179425
*Caloplacamonacensis*	Malíček 8255 (JM)	Ukraine	MG773668
*Caloplacastillicidiorum*	Gueidan s.n. (BCN)	France	EU639607
*Faurieachujaensis*	Kondratyuk SK D07 (KoLRI)	South Korea	KX793095
*Faurieaorientochinensis*	Wang & Hur SK710 (KoLRI)	China	KX793097
*Ioplacapindarensis*	Aptroot 56827 (ABL)	China	JQ301672
*Upretiaamarkantakana* 1	Kondratyuk SK E23 (LWG)	India	MG652763
*Upretiaamarkantakana* 2	Kondratyuk SK J21 (LWG)	India	MG652765
*Upretiaamarkantakana* 3	Kondratyuk SK J59 (LWG)	India	MG652766
*Upretiasquamulosa* 1	Wang et al. 17-56088 (KUN)	China	MH497054
*Upretiasquamulosa* 2	Wang et al. 15-47423 (KUN)	China	MH497055
*Upretiasquamulosa* 3	Wang et al. 16-50148 (KUN)	China	MH497056
*Upretiasquamulosa* 4	Wang et al. 13-41007 (KUN)	China	MH497059
*Upretiazeorina* 1	Wang et al. 19-63056 (KUN & FR)	China	**MW798796**
*Upretiazeorina* 2	Wang et al.19-63058 (KUN & FR)	China	**MW798795**
*Upretiazeorina* 3	Wang et al. 17-56125 (KUN)	China	**OP806864**
*Upretiazeorina* 4	Wang et al. 17-56127 (KUN)	China	**OP806866**
*Upretiazeorina* 5	Wang et al. 14-43393 (KUN)	China	**OP806863**
*Upretiazeorina* 6	Wang et al. 16-50177 (KUN)	China	**OP806865**
*Upretiazeorina* 7	Wang et al. 19-63045 (KUN)	China	**OP806871**
*Upretiazeorina* 8	Wang et al. 19-63040 (KUN)	China	**OP806870**
*Upretiazeorina* 9	Wang et al. 19-62891 (KUN)	China	**OP806868**
*Upretiazeorina* 10	Wang et al. 19-62896 (KUN)	China	**OP806869**
*Upretia* sp.1	Wang et al. 19-62841 (KUN)	China	**OP806862**
*Upretia* sp.2	Wang et al. 14-43454 (KUN)	China	**OP806867**

Phylogenetic reconstructions were carried out using maximum likelihood and Bayesian inference. Maximum likelihood phylogeny was inferred using the online version of IQ-TREE (http://iqtree.cibiv.univie.ac.at/, Trifinopoulos et al. 2016) with automated substitution model selection with three partitions (ITS1, 5.8S rDNA, ITS2). The best-fit model was selected according to the Bayesian information criterion (BIC) for individual per partition as: TNe+G4 for ITS1, K2P+I for 5.8S, TNe+I for ITS2. The Branch support was assessed using both ultrafast bootstrap approximation (UFBoot) (Minh et al. 2013) with 1000 replicates and the Shimodaira-Hasegawa-like approximate likelihood ratio test (SH-aLRT) (Guindon et al. 2010) with 1000 replicates. Nodes with support values of both UFBoot ≥ 95% and SH-aLRT ≥ 80% were considered as well-supported (Minh et al. 2013). Bayesian reconstruction of phylogeny was performed with MrBayes 3.2.6 (Ronquist et al. 2012) to infer phylogenetic trees applying the models inferred by ModelFinder (Kalyaanamoorthy et al. 2017) as: GTR+G for ITS1 and ITS2, K2P+I for 5.8S. Two parallel runs of four Markov chains each were run for 2 million generations, sampling every 1000^th^ generation, and the first 25% discarded as burn-in. The average standard deviation of split frequencies had fallen below 0.01 at the end of the analysis. SH-aLRT ≥ 80%, UFBoot ≥ 95% and Bayesian posterior probabilities ≥ 0.95 were visualized on the ML tree.

## ﻿Results and discussion

The nrITS dataset comprised 25 terminals, and 12 of them represented newly generated sequences in this study that were deposited in GenBank. All the reported species of *Upretia* with available sequences in GenBank were used in our study.

**Figure 1.**
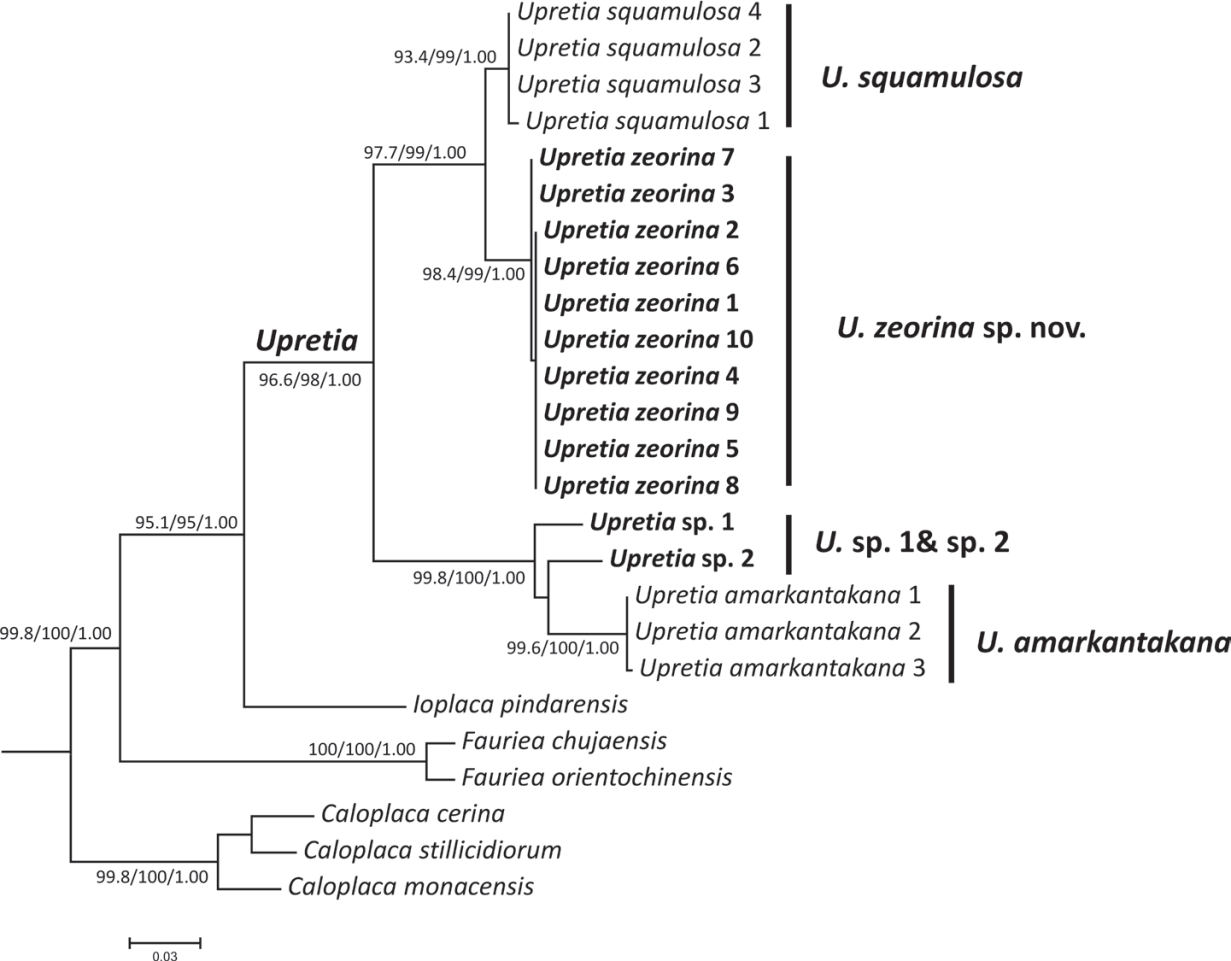
Phylogenetic tree generated from Maximum Likelihood based on nrITS sequences data. SH-aLRT support (%) ≥ 80 / ultrafastbootstrap support (%) ≥ 95 / Bayesian posterior probabilities (PPs) ≥ 0.95 are given above the nodes.

Phylogenetic inferences resolved the specimens of *Upretia* as a highly supported (SH-aLRT = 96.6%, UFBoot = 98%, PP = 1.00) monophyletic group (Fig. 1), sister grouped to *Ioplacapindarensis*. Ten of the newly collected Chinese specimens compose a robust clade (SH-aLRT = 98.4%, UFBoot = 99%, PP = 1.00) that is strongly supported as sister (97.7%, UFBoot = 99%, PP = 1.00) to the Chinese species *U.squamulosa*. Two other Chinese specimens (*Upretia* sp.1 and *Upretia* sp. 2) compose a grade subtending the Indian species *U.amarkantakana*, from which they differ in several phenotypic characters (see discussion below). In our preliminary phylogenetic analysis, we included all the available sequences of *Upretia* from GenBank. The type species *U.amarkantakana*, with four samples, appears paraphyletic at the base of *Upretia*, with *U.amarkantakana* SK J20 (ITS-MG652764, mtSSU-MG652767) (Kondratyuk et al. 2018) as basal to three further samples of *U.amarkantakana* and the remainder of the genus. The result is in agreement with Zhang et al. (2019) that the true phylogenetic position and delimitation of the species *U.amarkantakana* is not clear. Therefore, we exclude this sample from our final analysis here and do not show it in the phylogenetic tree (Fig. 1).

### ﻿Taxonomy

#### ﻿ Upretia zeorina

Taxon classificationFungiTeloschistalesTeloschistaceae

Li J. Li & Printzen sp. nov.

FB45579C-ABA2-56DA-BC54-5816BB104324

MycoBank No: 849819

Fig. 2A–F

##### Diagnosis.

Thallus epilithic, brown to blackish brown, areolate to squamulose, partly pruinose; apothecia zeorine type, disc black; ascospores polarilocular, 11.5–18.0 × 6.5–11.0 μm; conidia narrowly bacilliform, 4.0–6.0 × 0.5 μm. Containing gyrophoric acid.

**Figure 2.**
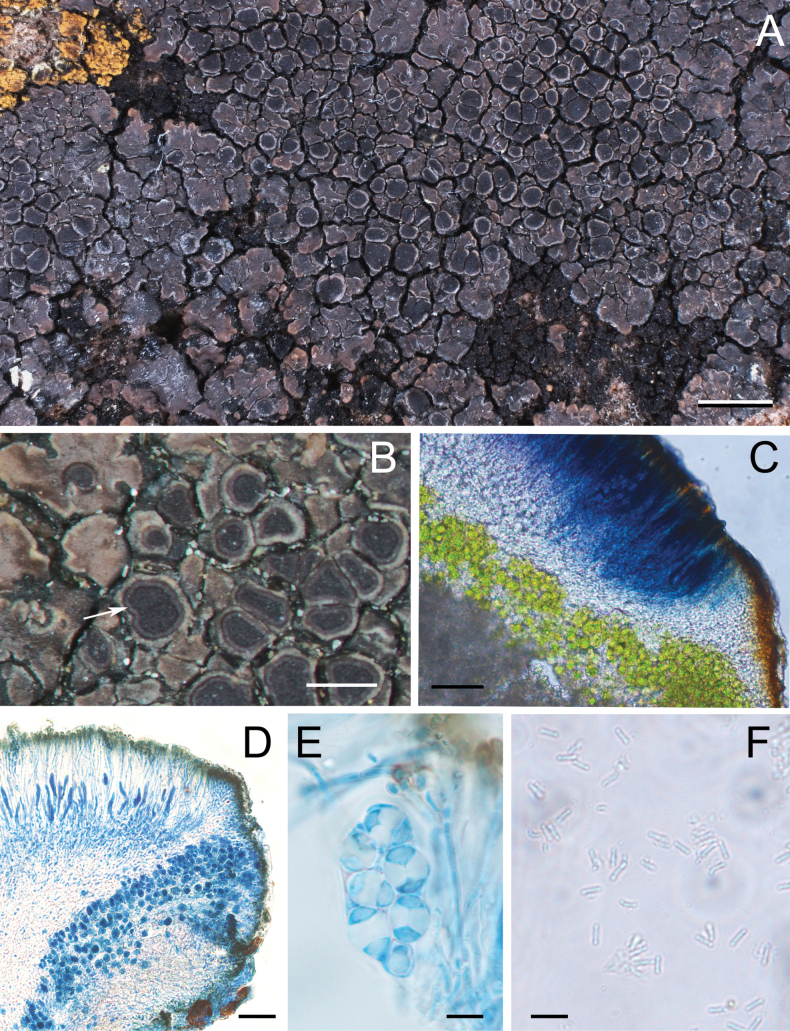
The new species *Upretiazeorina***A** habitus and lichen thallus **B** zeorine type apothecia, proper margin raised above disc (arrow) **C** section of apothecia in Lugol’s iodine (I), hymenium I+ blue, proper margin I- **D** section of apothecia in lactophenol cotton blue (LCB) **E** asci in lactophenol cotton blue, with polarilocular ascospores **F** shortly bacilliform conidia of the new species. Scale bars: 2 mm (**A**); 1 mm (**B**); 50 µm (**C**); 20 µm (**D**); 10 µm (**E, F**).

##### Type.

China. Sichuan Prov.: Huili Co., on the way from Huili to Jiaopingdu, elev. 1880 m, 26°21′N, 102°23′E, on rock, 11 Apr 2019, Wang Lisong et al. 19-63056 (KUN-L-66526–***Holotype***, FR-0183125–***Isotype***); GenBank No.: ITS-MW798796, mtSSU- MW798794.

##### Description.

Thallus areolate to squamulose, irregular in outline, squamules plane, adnate, rarely raised and free from substrate at edges, entire or rarely incised, 0.5–2.5 mm in diam. Upper surface brown to blackish brown, smooth or sparingly fissured, partly weakly shiny, ± white pruinose, mostly at the edges. Lower surface dark on the rising edge, without rhizines. Upper cortex brown, ca. 20 μm high; algal layer continuous, ca. 50–60 μm high, photobiont trebouxioid; medulla grey, ca. 120–160 μm high; lower cortex lacking.

Apothecia zeorine type, sessile, numerous, scattered to aggregated, rounded or irregular when aggregated, up to 1.2 mm in diam.; disc slightly concave to plane, black; proper margin persistent, slightly raised above or level with disc, brownish black, weakly shiny, consisting of interwoven hyphae, uppermost lateral part ca. 30–80 μm thick; thalline margin concolorous with the thallus, 30–130 μm thick, with olive cortical layer, 10–20 μm thick. Hymenium colorless, I+ blue, ca. 70–90 μm; epihymenium with brown pigment, 10–20 μm; paraphyses septate, rarely branched, ca. 2.0 μm wide, dark brown and swollen up to 4.0 μm at the tips; subhymenium and hypothecium colorless, 80–160 μm. Asci *Teloschistes*-type, 8-spored, 55–65 × 14–16 μm. Ascospores hyaline, polarilocular, ellipsoid to broadly ellipsoid, 11.5–18.0 × 6.5–11.0 μm, septum 6.0–9.0 µm. Pycnidia immersed, wall dark olive, conidia narrowly bacilliform, 4.0–6.0 × ca. 0.5 μm.

##### Chemistry.

Thallus and apothecia thalline margin K-, C+ red; HPTLC: only gyrophoric acid was detected.

##### Ecology and distribution.

**Figure 3.**
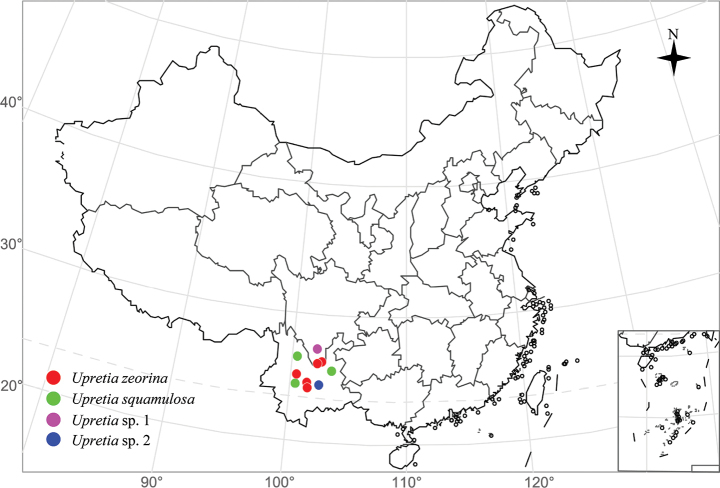
Locations of species of the genus *Upretia* in China. The map was obtained from National Platform for Common Geospatial Information Services (https://www.tianditu.gov.cn/).

On exposed rock in arid valley, at elevations between 1520 and 1880 m along the Jinsha-jiang River. Only known from Sichuan and Yunnan Provinces, China (Fig. 3).

##### Etymology.

Named due to its zeorine type of apothecia with a thalline outer and a proper inner margin.

##### Notes.

*Upretiaamarkantakana*, the type species of the genus *Upretia* known from India, differs from the new species by the olive-grey to brownish grey thallus with lobate margin, the lecanorine apothecia and orange to brown apothecial disc, the smaller ascospores (8.5–) 9.0–10.0 × 4.0–5.0 μm and shorter conidia (1.0–)2.0–3.0 × 0.5–1.0 μm, and the absence of gyrophoric acid (Joshi and Upreti 2006; Kondratyuk et al. 2018).

*Upretiasquamulosa* is similar to the new species with similar ascospores size (11.5–17.5 × 7.5–10.0 μm), squamulose thallus without lobate margin, the presence of gyrophoric acid, and similar distribution in Southwestern China (along the Jinshajiang River), but differs by having imbricate squamules with a non-pruinose, greyish green to brown upper surface, larger apothecia (up to 2 mm diam.) with a pale brown to brown disc, hyaline and slightly swollen paraphyses tips, and the presence of lecanoric acid (Zhang et al. 2019). Apothecia of *U.squamulosa* are usually lecanorine with the thalline margin at the early stage, and soon becoming zeorine, inner proper margin could be distinguished in sections, ca. 25–50 μm thick, thalline margin concolorous with the thallus, ca. 20–30 μm thick.

Molecular data are not available for *U.hueana*, and hence its phylogenetic relationships *U.zeorina* cannot be assessed, but the species distinctly differs by its squamulose to lobed thallus with brown surface, lecanorine apothecia with brown disc, smaller ascospores (8.5–11.0(–12.5) × 5.0–8.5 μm vs. 11.5–18.0 × 6.5–11.0 μm), the presence of parietin and the absence of gyrophoric acid (Wetmore 1996; Joshi and Upreti 2007; Mishra et al. 2020). As far as the currently known species in the genus *Upretia* are concerned, the Indian species differ from the Chinese species by their smaller ascospores, the secondary metabolites, and a preference for growing at lower altitudes (500–1050m) (Joshi and Upreti 2006; Kondratyuk et al. 2018; Mishra et al. 2020).

Mishra et al. (2020) mentioned that *Caloplacacupreorufa* Zahlbr., may belong to *Upretia* due to its brownish thallus and brownish pigment in walls of outer cortical cells. The species was collected by Handel-Mazzetti 1914 in Setschwan (Sichuan province, China) in a dry subtropical valley (Zahlbruckner 1930), close to the localities where the specimens of the new species were collected, but it differs by its smaller areoles (0.3–0.7 mm vs. 0.5–2.5 mm), its brown apothecial disc, lacking an inner proper margin, its apically unswollen and frequently branched paraphyses and its smaller ascospores (12.5–14.0 × 7.5–8.5 μm vs. 11.5–18.0 × 6.5–11.0 μm) (Zahlbruckner 1930; Wetmore 1994).

Two specimens of *Upretia* from Sichuan and Yunnan, i.e., *Upretia* sp. 1 and *Upretia* sp. 2 in figure 1, compose a clade with *U.amarkantakana*. They resemble the latter by their crustose thallus with lobate margins, but differ by the smaller areoles (0.2–0.5 mm) and shorter lobes (≤ 1.2 mm). *Upretia* sp.1 is characterized by the green thallus with partly pruinose on the margins, yellow medulla, and the presence of gyrophoric acid, whereas *Upretia* sp. 2 is characterized by a continuously pruinose upper surface, white medulla and the absence of gyrophoric acid. Unfortunately, only one sample has been collected each for these two putative species, respectively, and only the specimen of *Upretia* sp. 2 contains apothecia which are of the zeorine to lecanorine type. Therefore, we temporarily refrain from describing these two samples as new species until more populations and data are available. However, their morphological distinction within the genus *Upretia* highlights the further diversity of the genus in China, especially in the Hengduan Mountains.

##### Additional specimens examined.

*Upretiazeorina*: China. Sichuan Prov.: Huili Co., on the way from Huili to Jiaopingdu, elev. 1736–1880 m, 26°21′N, 102°19′E, on rock, 11 Apr 2019, Wang Lisong et al. 19-63058 (KUN-L-66528, FR- 0183126, mtSSU- MW798793), 19-63045 (KUN-L-66515), 19-63039 (KUN-L-66509), 19-63040 (KUN-L-66510), 19-63046 (KUN-L-66516), 19-62891 (KUN-L-66432), 19-62896 (KUN-L-66437); Yunnan Prov., Heqing Co., Zhongjiang Village, elev. 1540 m, 26°30′N, 100°23′E, on rock, 8 July 2016, Wang Lisong et al. 16-50177 (KUN-L-53525); Yuanmou Co., Langbapu Soil Forest, elev. 1526 m, 25°42′N, 101°41′E, on rock, 21 Apr 2014, Wang Lisong et al. 14-43393 (KUN-L-45200), on the way from Yuanmou to Yongren, elev. 1520 m, 25°58′N, 101°43′E, on rock, 1 July 2017, Wang Lisong et al. 17-56127(KUN-L-59563), 17-56125 (KUN-L-59561), 18-58007 (KUN-L-61584).

*Upretiasquamulosa*: China. Yunnan Prov.: Huize Co., Zhehai Town, elev. 1720m, 26°21′N, 102°19′E, on rock, 18 June 2015, Wang Xinyu et al. 15-47423 (KUN-L-50312-holotype, FR-0264988-isotype), 15-47427 (KUN-L-50316); Yulong Co., on the way from Lijiang to Ninglang, elev. 1871 m, 27°03′N, 100°30′E, on rock, 9 Apr 2019, Wang Lisong et al. 19-62704 (KUN-L-66245), Jiangbianxin Village, elev. 1720 m, 26°31′N, 103°42′E, on rock, 9 Dec 2013, Wang Lisong et al. 18-58077 (KUN-L-61568).

*Upretia* sp. 1: China. Sichuan Prov.: Dechang Co., on the way from Dechang to Huili, elev. 1320 m, 27°18′N, 102°19′E, on rock, 11 Apr 2019, Wang Lisong et al. 19-62841 (KUN-L-66382).

*Upretia* sp. 2: China. Yunnan Prov.: Yunlong Vil., Yunlong water reservoir, elev. 2100 m, 25°51′N, 102°22′E, on rock, 18 Apr 2014, Wang Lisong et al. 14-43454 (KUN-L-45260).

### ﻿World key to species of *Upretia*

**Table:** 

1	Thallus distinctly lobate at the margin	**2**
–	Thallus without a lobate margin	**4**
2	Lobes long, (0.5–)1.5–2.5(–3.5) mm in length, upper surface olive-grey to brownish grey, partly pruinose	***U.amarkantakana***
–	Lobes shorter than 1.2 mm in length, known from Southwestern China	**3**
3	Thallus partly pruinose on the margins, medulla yellow, producing gyrophoric acid	***U.* sp. 1**
–	Thallus totally pruinose on the upper surface, medulla withe, with zeorine to lecanorine type apothecia, lacking gyrophoric acid	***U.* sp. 2**
4	Thallus blackish brown, partially with pruina, apothecial disc zeorine-type, blackish	***U.zeorina***
–	Thallus brownish, without pruina, apothecial disc lecanorine-type, brown	**5**
5	Apothecia 0.5–2.5 mm diam., ascospores 11.5–17.5 × 7.5–10.0 μm, producing lecanoric acid in addition to gyrophoric acid, only known from Southwestern China	***U.squamulosa***
–	Apothecia 0.3–1.0 mm diam., ascospores 8.5–11.0(–12.5) × 5.0–8.5 μm, only producing parietin instead of gyrophoric acid, known from Mexico and India	***U.hueana***

## Supplementary Material

XML Treatment for Upretia zeorina

## ﻿Additional information

### Author ORCIDs

Lijuan Li https://orcid.org/0000-0003-1048-1971

Yanyun Zhang https://orcid.org/0000-0002-0902-5066

Christian Printzen https://orcid.org/0000-0002-0871-0803

Lisong Wang https://orcid.org/0000-0003-3721-5956

Xinyu Wang https://orcid.org/0000-0003-2166-6111

### Data availability

All of the data that support the findings of this study are available in the main text.
